# Ceftazidime–avibactam versus polymyxins in treating patients with carbapenem-resistant Enterobacteriaceae infections: a systematic review and meta-analysis

**DOI:** 10.1007/s15010-023-02108-6

**Published:** 2023-10-25

**Authors:** Jinglan Chen, Qin Hu, Pengxiang Zhou, Sheng Deng

**Affiliations:** 1 Department of Pharmacy, Xiangya Hospital, Central South University, Changsha, 410008 Hunan People’s Republic of China; 2 Department of Pharmacy, The First Affiliated Hospital of Guangxi Medical University, Nanning, 530000 Guangxi Zhuang Autonomous Region People’s Republic of China; 3 Institute for Rational and Safe Medication Practices, National Clinical Research Center for Geriatric Disorders, Xiangya Hospital, Central South University, Changsha, 410008 Hunan People’s Republic of China; 4 Department of Pharmacy, Peking University Third Hospital, Beijing, 100191 People’s Republic of China; 5 Institute for Drug Evaluation, Peking University Health Science Center, Beijing, 100191 People’s Republic of China; 6 Hospital Institute Administration, Central South University, Changsha, 410008 Hunan People’s Republic of China

**Keywords:** Carbapenem-resistant Enterobacteriaceae, CRE, Ceftazidime–Avibactam, Polymyxins, Meta-analysis

## Abstract

**Objective:**

Carbapenem-resistant Enterobacteriaceae (CRE) pose a significant threat to human health and have emerged as a major public health concern. We aimed to compare the efficacy and the safety of ceftazidime–avibactam (CAZ–AVI) and polymyxin in the treatment of CRE infections.

**Methods:**

A systematic review and meta-analysis was performed by searching the databases of EMBASE, PubMed, and the Cochrane Library. Published studies on the use of CAZ–AVI and polymyxin in the treatment of CRE infections were collected from the inception of the database until March 2023. Two investigators independently screened the literature according to the inclusion and exclusion criteria, evaluated the methodological quality of the included studies and extracted the data. The meta-analysis was performed using RevMan 5.4 software.

**Results:**

Ten articles with 833 patients were included (CAZ–AVI 325 patients vs Polymyxin 508 patients). Compared with the patients who received polymyxin-based therapy, the patients who received CAZ–AVI therapy had significantly lower 30-days mortality (RR = 0.49; 95% CI 0.01–2.34; *I*^2^ = 22%; *P* < 0.00001), higher clinical cure rate (RR = 2.70; 95% CI 1.67–4.38; *I*^2^ = 40%; *P* < 0.00001), and higher microbial clearance rate (RR = 2.70; 95% CI 2.09–3.49; *I*^2^ = 0%; *P* < 0.00001). However, there was no statistically difference in the incidence of acute kidney injury between patients who received CAZ–AVI and polymyxin therapy (RR = 1.38; 95% CI 0.69–2.77; *I*^2^ = 22%; *P* = 0.36). In addition, among patients with CRE bloodstream infection, those who received CAZ–AVI therapy had significantly lower mortality than those who received polymyxin therapy (RR = 0.44; 95% CI 0.27–0.69, *I*^2^ = 26%, *P* < 0.00004).

**Conclusions:**

Compared to polymyxin, CAZ–AVI demonstrated superior clinical efficacy in the treatment of CRE infections, suggesting that CAZ–AVI may be a superior option for CRE infections.

**Supplementary Information:**

The online version contains supplementary material available at 10.1007/s15010-023-02108-6.

## Introduction

In recent decades, carbapenem-resistant Enterobacteriaceae (CRE) have been identified by the World Health Organization as a formidable medical threat to public health [1, 2]. Infections caused by CRE resulted in extremely high morbidity and mortality, and recent studies have revealed a mortality rate exceeding 65% for bloodstream infections (BSIs) caused by CRE [3]. Despite the severity of CRE infections, the treatment options for CRE infections are still very limited. Currently, carbapenems (meropenem, imipenem), polymyxins, tigecycline, aminoglycosides, and ceftazidime–avibactam (CAZ–AVI) are the main antibiotics used to treat CRE infections [4, 5]. Based on reports of in vitro activity and clinical efficacy, polymyxins has been used as a first-line agent to treat CRE infections [6, 7].

The polymyxins currently in clinical use include colistin and polymyxin B [8]. However, due to its toxicity (such as nephrotoxicity and neurotoxicity), limited efficacy, dose uncertainty (suboptimal pharmacokinetic dose), heterogeneous resistance mediated by mcr-1, and the limited accuracy of in vitro susceptibility testing, polymyxin cannot be utilized as a last resort for the treatment of CRE infections [6, 9–12].

In 2015, CAZ–AVI was approval by the U.S. Food and Drug Administration (FDA) for the treatment of complicated abdominal infections (cIAIs), complicated urinary tract infections (cUTIs), as well as hospital-acquired, and ventilator-associated pneumonia (HAP/VAP) [13]. In 2019, CAZ–AVI was approved in China as the only new β-lactam/β-lactamase inhibitor combinations for the treatment of cIAI, HAP, and VAP caused by multi-drug-resistant Gram-negative bacteria. Preliminary evidence suggests that CAZ–AVI-based regimens are more effective than current treatments for CRE infections [14–16].

Due to the limited availability of data, previous meta-analyses on the efficacy of CAZ-AVI in treating CRE infections usually paid little attention to the comparison of CAZ–AVI with specific antibacterial agents. Meta-analyses have been conducted to compare the efficacy of CAZ–AVI with other available antibacterial agents, including the mix of carbapenems, polymyxins, tigecycline, and aminoglycosides [17, 18]. However, no meta-analysis has been conducted to compare the efficacy of CAZ-AVI and polymyxins in treating CRE infection, and most of the real-world studies comparing antibacterial agents for the treatment of CRE have been small sample sizes. The aim of this meta-analysis was therefore to compare the efficacy of CAZ–AVI-based therapy with polymyxin-based therapy in the treatment of CRE infections.

## Methods

### Search strategy

This study was conducted according to PRISMA statement. A systematic search was conducted across three databases, namely PubMed, EMBASE, and the Cochrane Library. Additionally, a manual search of references were performed to identify included literature. The search period spans from the inception of database construction to March 18, 2023. The search terms utilized were “carbapenem-resistant Enterobacteriaceae”, “avibactam-ceftazidime”, and “polymyxins”. Subject headings and free texts (i.e., Medical Subject Headings [MeSH] terms) were identified for the search terms. The complete search strategies are provided in the Supplementary Material.

### Selection criteria

Two investigators independently conducted the literature search and screened the literature. Studies were eligible for inclusion if they (1) compared the efficacy of CAZ-AVI and polymyxins in patients with CRE infections, (2) reported primary outcome (30-days mortality), and (3) were prospective/retrospective observational cohort, case–control studies, or randomized controlled trials (RCTs), Exclusion criteria were (1) studies not published in English, (2) studies that did not provide adequate information.

### Quality assessment

The literature's quality was independently evaluated by two investigators. The methodological quality of cohort or case–control studies was assessed using the Newcastle–Ottawa Scale (NOS), including risk of bias in patient selection, comparability between groups, and exposure or outcome. Studies with NOS scores ≥ 7 were high quality studies. The methodological quality of included RCTs was assessed using the Cochrane Collaboration ‘risk of bias’ tool.

### Data extraction

The following information was extracted from the included studies: (1) first author and publication year, (2) study characteristics including design, duration, and sample size, (3) patient characteristics including infection type and pathogen, and (4) clinical outcomes: 30-days mortality rate, clinical cure rate, microbial clearance rate, and adverse effects rate.

### Definitions

The primary outcome of this study was 30-days mortality including 28-days mortality, while the secondary outcomes were clinical cure and microbial clearance. Clinical cure was defined as the resolution of clinical signs and symptoms of infection, as documented by the clinician, along with follow-up data indicating that the patient has achieved microbiological eradication [19]. Microbial clearance was defined as negative CRE culture after antibacterial therapy. CAZ–AVI-based therapy was defined as treatment with CAZ–AVI or in combination with other antibacterial agents. Polymyxin-based therapy was defined as treatment with polymyxin or in combination with other antimicrobial agents other than CAZ–AVI [20].

### Statistical analysis

Meta-analysis was performed using Review Manager 5.3 statistical software provided by the Cochrane Collaboration network. All outcome indicators in this study were dichotomous variables, so relative risk (RRs) and 95% confidence interval (CI) were used to express them. Testing for heterogeneity using the *I*^2^ statistic, random effects models were used for results with high heterogeneity (*I*^2^ > 50%), while fixed effects models were used when heterogeneity was not significant. Inverted Funnel plot was used to detect publication bias, and sensitivity analysis was used to determine the robustness of the results of this analysis.

## Results

### Study selection

**Fig. 1:**
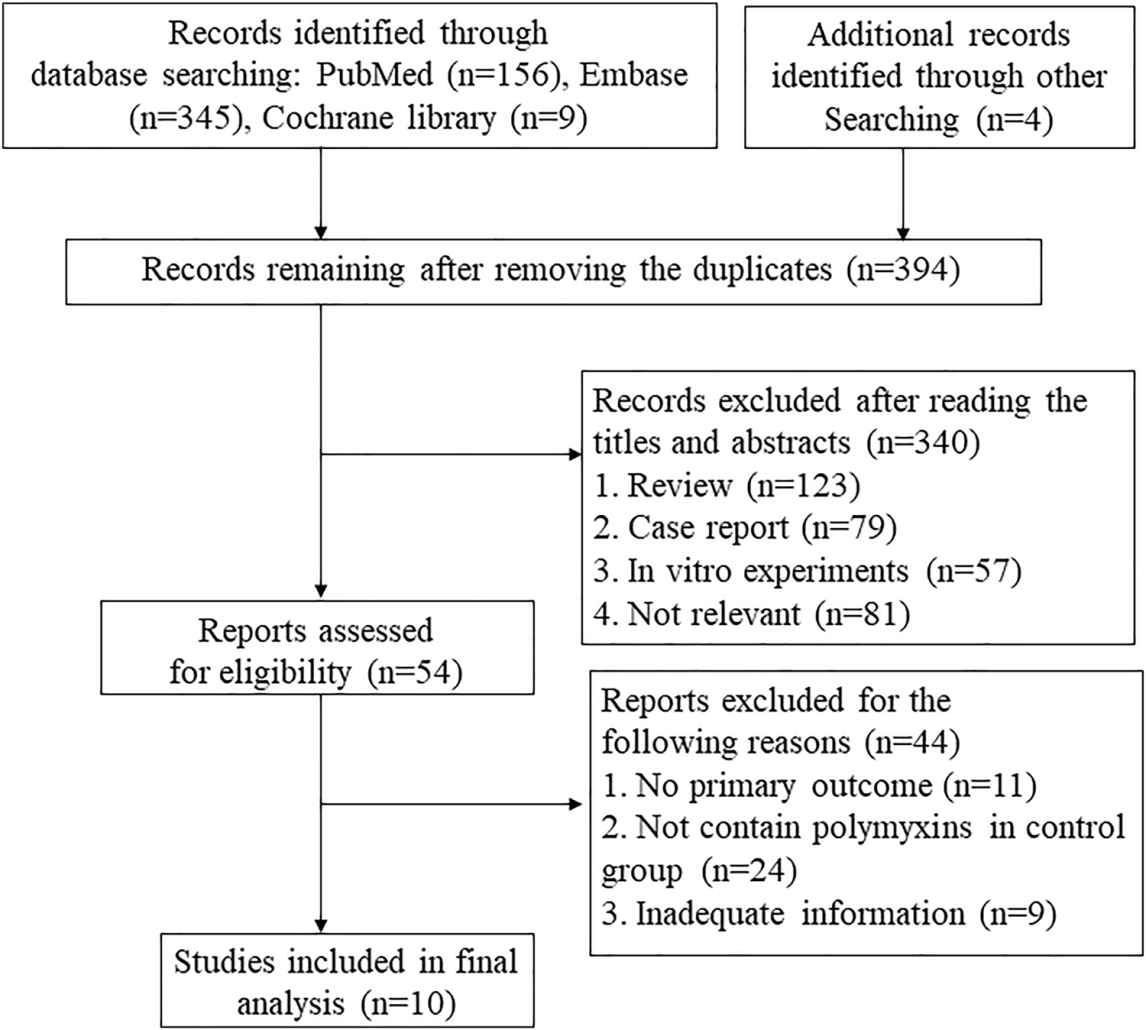
Flow diagram of included studies

The search strategy developed in this study yielded 510 references through electronic database search and an additional 4 references were identified via reference list. There were 394 references after removing duplicates, and 340 references were excluded after reading the abstract and title. Of the 54 available literatures, eleven were excluded due to lack of reported primary outcomes, 24 were excluded because they did not include polymyxin in the control group, and 9 were excluded due to unavailability of important data. Finally, ten references (nine full paper and one conference paper) were included in this study for meta-analysis. Figure 1 listed the flow diagram of included studies.

### Study characteristics

The characteristics of the included ten studies are listed in Table 1. In ten studies [21–30], all were observational studies, eight of which were retrospective, and two of which were prospective, including two case–control, eight cohort studies. Among the ten studies, six were multicenter studies, and four were single-center studies. The sample size ranged from 32 to 164. The included ten studies enrolled 833 patients with 325 receiving the CAZ–AVI therapy and 508 receiving the polymyxin therapy. Table 1 lists the specific antimicrobial regimens for each study and their effects on 30-day mortality. All ten studies reported 30-day mortality (or 28-day mortality), three studies reported clinical cure rates, two studies reported microbial clearance, and three studies reported acute kidney injury. The most frequently investigated pathogen was CRKP, followed by CR and CPE. The primary infections were BSIs, followed by any infection (Table 1). The scoring details are shown in Table 2. The NOS scores of ten studies were ≥ 7 scores (Table 2).

**Table 1:** Characteristics of the included studies

Study	Design	Sample size	Population	Study period	Pathogen	Infection type	CAZ–AVI-based regimens (30-day mortality, *n*, %)	Polymyxin-based regimens (30-day mortality, *n*, %)	Outcome
Chen 2021	R, case–control/MC	138	In patients	2018–2020	CRE	BSI	CAZ-AVI (3, 23.1); CAZ-AVI + Tige (2, 15.4); CAZ-AVI + Tige + Poly (1, 11.1)	Poly B + Tig (19, 41.3); Poly B + Tig + Carb (16, 36.4); Poly B + Carb + amino (5, 38.5)	30-day mortality
Zheng 2020	R, cohort/MC	164	Critically ill patients	2019–2021	*CRKP*	BSI, RTI, IAI, UTI	CAZ-AVI monotherapy (42, 51.5); CAZ-AVI combination (20, 24.5)	Poly B-based (25, 30.5)	30-day mortality; Microbial clearance
Shields 2017	R, cohort/SC	43	In patients	2009–2017	*CRKP*	BSI	CAZ-AVI monotherapy (1, 7.7)	COL + Carb (13, 43.3)	30-day mortality; Clinical cure
Meng 2022	R, case–control/SC	37	Hematological malignancy	2018–2021	*CRKP*	BSI	CAZ-AVI monotherapy (1, 14.3)	Poly B monotherapy (22, 73.3)	28-day mortality
Hakeama 2021	R, cohort/MC	61	In patients	2017–2020	CRE	BSI	CAZ-AVI-based (12, 37.5)	COL-based combination (12, 41.4)	30-day mortality; Clinical cure; Acute kidney injury
Satlin 2022	R, cohort/MC	47	In patients	2016–2018	CRE	BSI	CAZ-AVI monotherapy (2, 10)	Poly B monotherapy (8, 31)	30-day mortality; Acute kidney injury
Falcone 2021	P, cohort/SC	79	In patients	2018–2019	*CPE*	BSI	CAZ-AVI + ATM (10, 19.2)	COL monotherapy (1, 50); COL-based combination (15, 57.7)	30-day mortality
Zhou 2021	P, cohort/MC	32	In patients	2019	CRE	BSI	CAZ-AVI monotherapy (0, 0); CAZ-AVI combination (0, 0);	Poly B monotherapy (1, 33.3); Poly B combination (16, 64)	30-day mortality
Fang 2021	R, cohort/MC	115	In patients	2018–2020	CRE	BSI, Pneumonia, IAI	CAZ-AVI-based (3, 8.1)	Poly B-based (12, 29.5)	28-day mortality; Clinical cure; Microbial clearance; Acute kidney injury
John 2019	R, cohort/SC	117	In patients	2010–2018	CRE	BSI, RTI, UTI	CAZ-AVI-based (9, 21.4)	Poly B-based (19, 25.3)	30-day mortality

*P* prospective, *R* retrospective, *MC* multicenter, *SC* single center, *CRE* carbapenem-resistant Enterobacteriaceae, *CRKP* carbapenem-resistant *Klebsiella pneumoniae*, *CPE* carbapenemase–producing Enterobacteriaceae, *BSI* bloodstream infection, *RTI* Respiratory tract infection, *IAI* Intra-abdominal infection, *UTI* Urinary tract infection, *Tige* Tigecycline, *Poly B* Polymyxin B, *Carb* carbapenem, *amino* aminoglycoside, *COL* colistin, *ATM* Aztreonam

**Table 2:** Quality assessment

Risk of bias for cohort studies									
Study	Selection				Comparability	Exposure			Total Score
	Exposed cohort	Non-exposed cohort	Ascertainment of exposure	Outcome of interest		Assessment of outcome	Length of follow-up	Adequacy of follow-up	
Zheng 2020	0	1	1	0	2	1	1	1	7
Shields 2017	1	1	1	0	1	1	1	1	7
Hakeama 2021	1	1	1	0	1	1	1	1	8
Satlin 2022	1	1	1	0	1	1	1	1	7
Falcone 2021	1	1	1	1	1	1	1	1	8
Zhou 2021	1	1	1	1	1	1	1	1	8
Fang 2021	1	1	1	0	1	1	1	1	7
John 2019	1	1	1	0	1	1	1	1	7

Risk of bias for case–control studies									
Study	Selection				Comparability	Exposure			Total Score
	Definition of the case	Representativeness of the cases	Selection of controls	Definition of controls		Ascertainment of exposure	Method of ascertainment	Non-Response rate	
Chen 2021	1	1	0	1	2	1	1	1	8
Meng 2022	1	1	0	1	2	1	1	1	8

### Results of meta-analysis

**Fig. 2:**
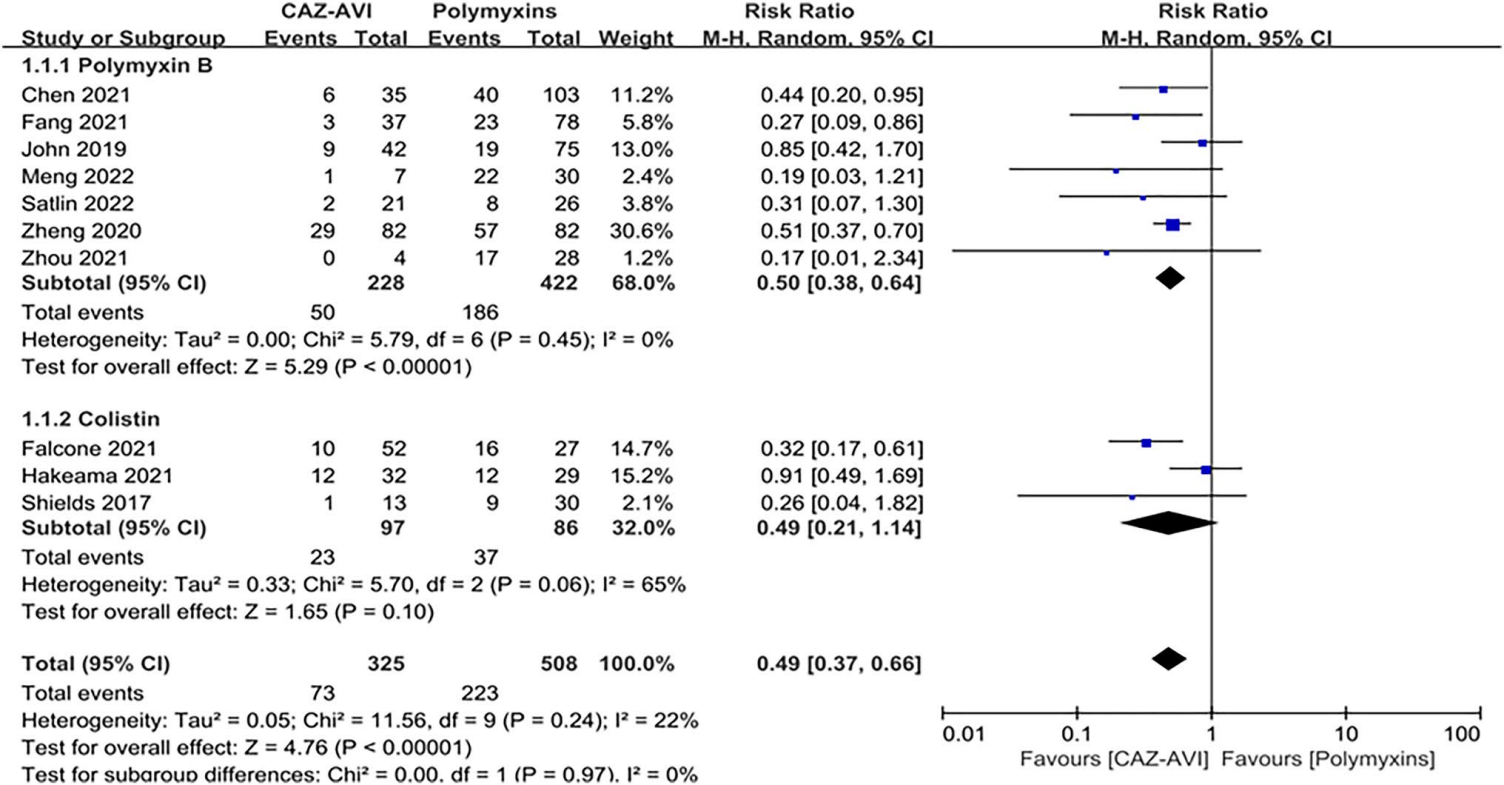
Thirty-day mortality of the CAZ–AVI-based therapy compared with polymyxin-based therapy in CRE infections

All 10 studies reported 30-day mortality. Compared with the patients who received polymyxin-based therapy, the patients who received CAZ–AVI therapy had significantly lower 30-day mortality (RR = 0.49; 95% CI 0.01–2.34;* I*^2^ = 22%; *P* < 0.00001; Fig. 2). Subgroup analysis showed that 30-day mortality was significantly lower in patients treated with CAZ–AVI than in patients treated with polymyxin B (RR = 0.50; 95% CI 0.38 ~ 0.64; *I*^2^ = 0%; *P* < 0.00001; Fig. 2), while 30-day mortality was lower in patients treated with CAZ–AVI than in patients treated with colistin, but there was no statistical difference (RR = 0.49; 95% CI 0.21 ~ 1.14; *I*^2^ = 65%; *P* < 0.10; Fig. 2).

**Fig. 3:**
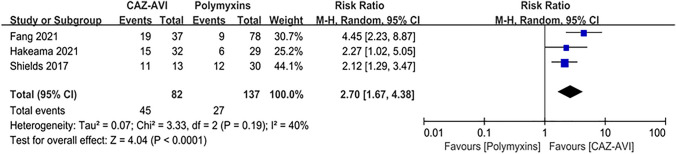
Clinical cure of the CAZ-AVI-based therapy compared with polymyxin-based therapy in CRE infections

**Fig. 4:**
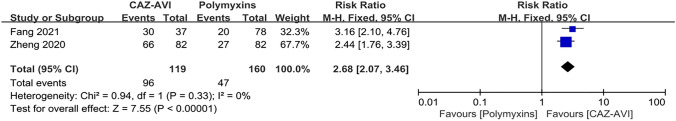
Microbial clearance of the CAZ-AVI-based therapy compared with polymyxin-based therapy in CRE infections

**Fig. 5:**
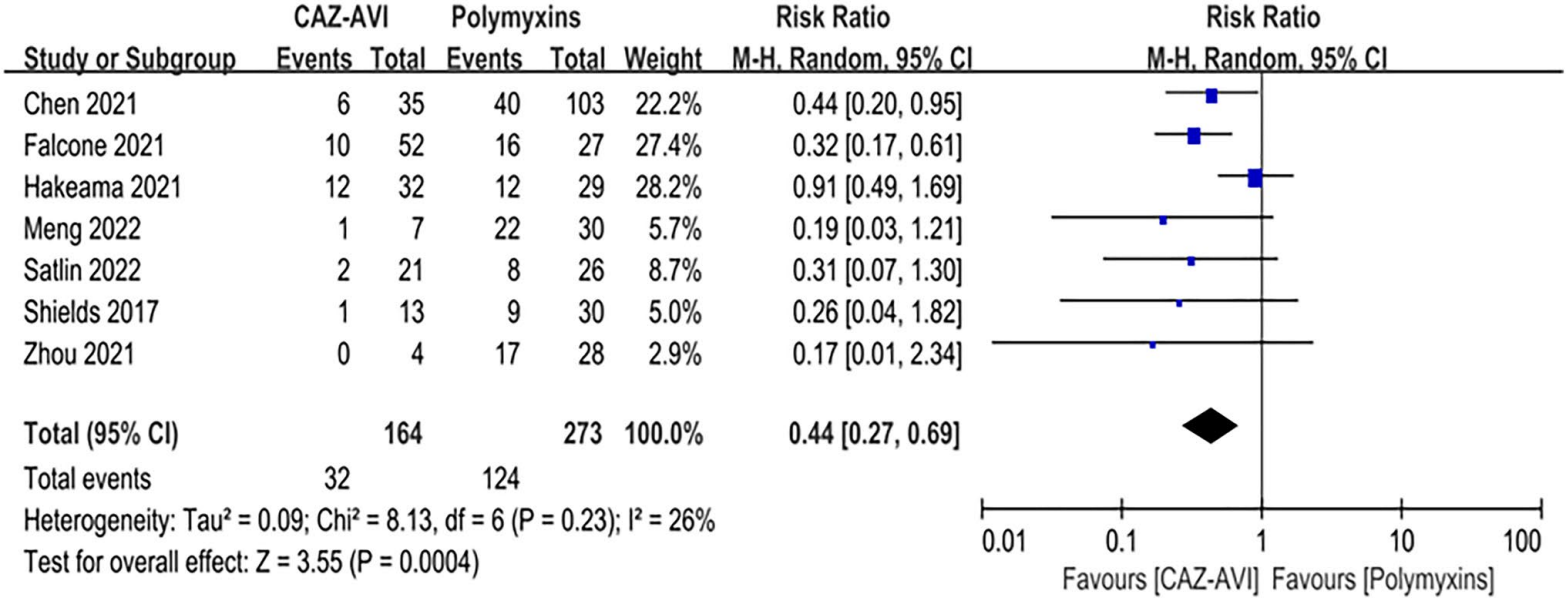
Thirty-day mortality of the CAZ–AVI-based therapy compared with polymyxin-based therapy in CRE BSI

**Fig. 6:**
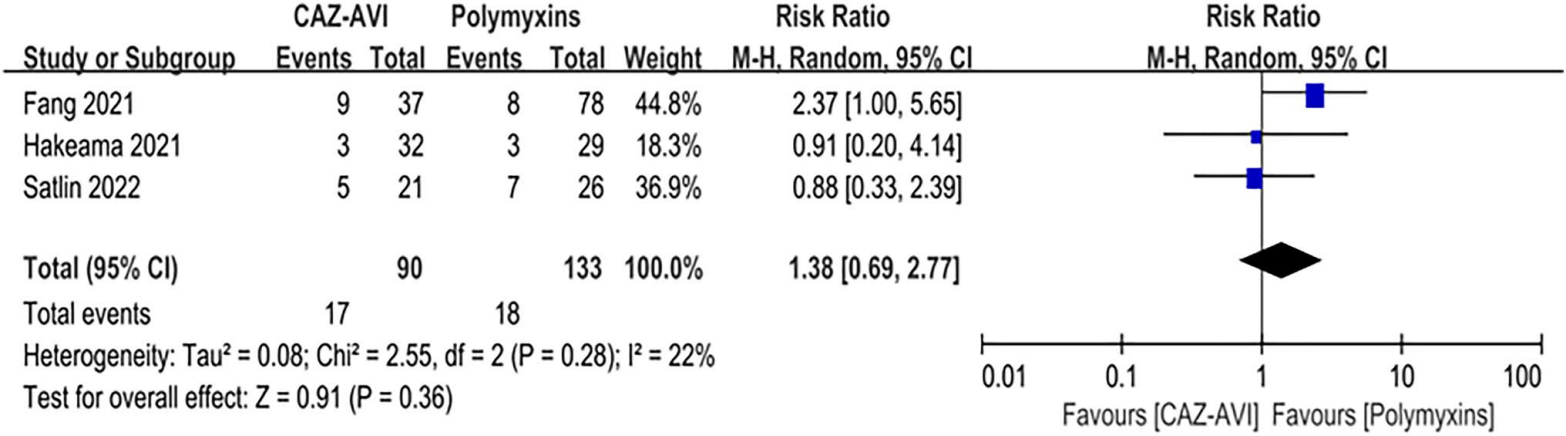
Acute kidney injury of the CAZ–AVI based therapy compared with polymyxin-based therapy in CRE infections

Compared with the patients who received polymyxin-based therapy, the patients who received CAZ–AVI therapy had significantly higher clinical cure rate (RR = 2.70; 95% CI 1.67 ~ 4.38; *I*^2^ = 40%; *P* < 0.00001; Fig. 3), and higher microbial clearance rate (RR = 2.70; 95% CI 2.09 ~ 3.49; *I*^2^ = 0%; *P* < 0.00001, Fig. 4). In addition, among patients with CRE bloodstream infection, those who received CAZ–AVI therapy had significantly lower mortality than those who received polymyxin therapy (RR = 0.44; 95% CI 0.27 ~ 0.69, *I*^2^ = 26%, *P* < 0.00004; Fig. 5). However, there was no statistically difference in the incidence of acute kidney injury between patients who received CAZ–AVI and polymyxin therapy (RR = 1.38; 95% CI 0.69 ~ 2.77; *I*^2^ = 22%; *P* = 0.36; Fig. 6).

### Sensitivity analysis

Sensitivity analysis was performed by sequentially excluding studies in this meta-analysis. The finally results demonstrated no significant alterations in RR, *P*, and* I*^*2*^ outcomes following the exclusion of each study. The sensitivity analysis did not have an impact on the 30-day mortality, clinical cure rate, microbial clearance, and incidence of acute kidney injury, which indicates that the results of this meta-analysis have certain robustness.

## Discussion

The options for treating CRE infections with antibiotics are extremely limited. In the clinical management of CRE infection, the selection of appropriate antibiotics has always posed a formidable challenge. Early selection of appropriate active antibiotics following infection with CRE is crucial for reducing mortality rates and improving clinical outcomes. According to the Infectious Diseases Society of America (IDSA) and the European Society for Clinical Microbiology and Infectious Diseases (ESCMID), the optimal antimicrobial regimen for treating CRE infections remains undetermined [4, 5].

The results of this meta-analysis indicated CAZ–AVI-based therapy was significantly superior to polymyxin-based therapy. Furthermore, the findings of this study demonstrate that CAZ–AVI outperforms polymyxin in terms of the primary outcome for patients with CRE-BSI.

Prior to the introduction of new drugs, such as CAZ–AVI, polymyxin was frequently utilized in both monotherapy and combination regimens. However, the following limitations limit the use of polymyxins: (1) Polymyxin exhibits high incidence of adverse reactions, particularly nephrotoxicity and neurotoxicity, (2) Polymyxins are antibacterial drugs that exhibit concentration-dependent activity and have a relatively narrow therapeutic window. For instance, polymyxin E achieves an effective steady-state blood concentration of 2 mg/L, with the risk of nephrotoxicity increasing at concentrations exceeding 2.3 mg/L—a range that almost overlaps with the threshold for toxicity, (3) Both colistin and polymyxin B can induce drug resistance during treatment; thus, combination therapy is recommended for severe infections, (4) It is difficult to reach the required concentration of the drug in lung tissue and body fluids during intravenous administration of polymyxin, and the PK/PD target achievement rate in patients with pulmonary infection is significantly decreased [10–12]. The results of this study indicate that there was no significant difference in the incidence of severe kidney injury between patients treated with polymyxin and those treated with CAZ–AVI. This may be attributed to insufficient attention paid to the comparison of nephrotoxicity between CAZ–AVI and polymyxin, resulting in inadequate sample size and inaccurate findings.

In recent years, CAZ–AVI, as a new antibacterial combination, was approved by the US FDA for the treatment of CRE infection in 2015. Studies have analyzed the effect of CAZ–AVI-based therapy on adverse outcomes in patients with CRE infections. The study findings indicated that there was no statistically significant difference in mortality rates between patients who received combination therapy with CAZ–AVI and those who received monotherapy [16, 30, 31]. This conclusion was further supported by two separate meta-analyses [32, 33]. Similarly, many studies have compared the efficacy of CAZ–AVI with other antibacterial agents, including carbapenems, tigecycline, and polymyxin for treating infections caused by CRE. The results showed a higher mortality rate in patients treated with other antimicrobial agents [15, 16, 28, 31, 34]. This finding was also supported by several meta-analyses [17, 18, 35]. CAZ-AVI in combination with another in vitro-sensitive antimicrobial agent, including carbapenems, fosfomycin, or tigecycline, significantly reduced 30-day mortality in critically ill patients with CRE infections [14]. However, a larger sample size is required to validate this conclusion and to identify more optimal antimicrobial agents for combination therapy regimens. In short, preliminary evidence suggests a potential role for CAZ–AVI in patients with CRE infection.

However, the gradual increase in resistance to CAZ–AVI has resulted in reduced efficacy due to β-lactamase production, efflux pump activity and target modification [36]. In addition to CAZ–AVI, other novel antibiotics for the treatment of CRE infections have been approved or are in advanced clinical development, including ceftolozane–tazobactam, meropenem–vaborbactam, and imipenem–cilastatin–relebactam [37]. Meropenem–vaborbactam has demonstrated promising outcomes in treating CRE infections in the TANGO II clinical trial. However, given its limited sample size, further clinical studies are necessary to evaluate both its efficacy and safety. Due to limited data on these novel antibiotics, we did not compare the efficacy of these antimicrobials to that of CAZ–AVI or other antimicrobials. As the prevalence of drug-resistant continues to escalate, there is an urgent need for the development of novel therapeutics to combat infections caused by CRE.

This study has several limitations, first, the included studies were observational studies with small sample sizes and no randomized controlled trials (RCTs), which inevitably introduces confounding factors and bias; Second, the heterogeneity of colistin was found to be greater in the subgroup analysis. The study design and the pathogens were identified as potential reasons for this variability; Third, only three studies have reported data on the nephrotoxicity of CAZ–AVI and polymyxin, with no other adverse reactions such as neurotoxicity or cutaneous adverse reactions being reported. Finally, due to limited data, this study did not control for other confounding factors (such as the severity of patients’ infections, underlying diseases, etc.).

## Conclusions

The meta-analysis compared the efficacy and the safety of CAZ–AVI and polymyxins in the treatment of CRE infections. Compared to polymyxins, CAZ–AVI demonstrated superior clinical efficacy in the treatment of CRE infections, suggesting that CAZ–AVI may be a superior option for CRE infections. In addition, there was no significant difference in safety between the two treatment options.

### Supplementary Information

Below is the link to the electronic supplementary material.

Supplementary file1 (DOCX 17 KB)

## Data Availability

The authors confirm that the data supporting the findings of this study are available within the article [and/or supplementary materials].
