# Supplementary material for: Ceftazidime–avibactam versus polymyxins in treating patients with carbapenem-resistant Enterobacteriaceae infections: a systematic review and meta-analysis
Source: Infection. 2023 Oct 25;52(1):19–28. doi: 10.1007/s15010-023-02108-6 (PMC10810944; doi:10.1007/s15010-023-02108-6)
Supplement: Supplementary file 1 — Supplementary file1 (DOCX 17 KB) [file 15010_2023_2108_MOESM1_ESM.docx]

**Supplementary Material**

**Ceftazidime-Avibactam Therapy Versus Polymyxin Therapy in Patients with Carbapenem-resistant Enterobacteriaceae Infections: A Systematic Review and Meta-Analysis**

**1. Search Strategy**

**PubMed**

((("Carbapenem-Resistant Enterobacteriaceae"[Mesh]) OR ((Carbapenemase-Producing Enterobacteriaceae[Title/Abstract]) OR (carbapenem resistant Klebsiella pneumoniae[Title/Abstract]) OR (carbapenem resistant Escherichia coli[Title/Abstract]) OR (Antibiotics, Carbapenem[Title/Abstract]) OR (Carbapenem Antibiotics[Title/Abstract]) OR (carbapenem resist[Title/Abstract]) OR (carbapenemase producing[Title/Abstract]) OR (carbapenem resistance[Title/Abstract]) OR (carbapenem non-susceptible Enterobacteriaceae[Title/Abstract]) OR (CNSE (Enterobacteriaceae)[Title/Abstract]) OR (CRE (Enterobacteriaceae)[Title/Abstract]) OR (carbapenem nonsusceptible enterobacterial isolate[Title/Abstract]) OR (Carbapenem-Resistant Enterobacterales[Title/Abstract]))) AND (("avibactam, ceftazidime drug combination" [Supplementary Concept]) OR ((avibactam-ceftazidime[Title/Abstract]) OR (ceftazidime-avibactam[Title/Abstract]) OR (Avycaz[Title/Abstract]) OR (avibactam plus ceftazidime[Title/Abstract]) OR (nxl104[Title/Abstract]) OR (ave1330a[Title/Abstract]) OR (CAZ-AVI[Title/Abstract])))) AND (("Polymyxins"[Mesh]) OR ((Polymyxin[Title/Abstract]) OR (Polymyxin B[Title/Abstract]) OR (Polymyxin M[Title/Abstract]) OR (Colistin[Title/Abstract]) OR (Polymyxin E[Title/Abstract]) OR (Colimycin[Title/Abstract]) OR (Colisticin[Title/Abstract]) OR (Coly-Mycin[Title/Abstract]) OR (Totazina[Title/Abstract]) OR (Colistin Sulfate[Title/Abstract]) OR (Sulfate, Colistin[Title/Abstract])))

**Embase**

#1 'carbapenem-resistant enterobacteriaceae'/exp 4444

#2 'carbapenemase-producing enterobacteriaceae':ab,ti,kw OR 'carbapenem resistant klebsiella pneumoniae':ab,ti,kw OR 'carbapenem resistant escherichia coli':ab,ti,kw OR 'antibiotics, carbapenem':ab,ti,kw OR 'carbapenem antibiotics':ab,ti,kw OR 'carbapenem resist':ab,ti,kw OR 'carbapenemase producing':ab,ti,kw OR 'carbapenem resistance':ab,ti,kw OR 'carbapenem non-susceptible enterobacteriaceae':ab,ti,kw OR 'cnse (enterobacteriaceae)':ab,ti,kw OR 'cre (enterobacteriaceae)':ab,ti,kw OR 'carbapenem nonsusceptible enterobacterial isolate':ab,ti,kw OR 'carbapenem-resistant enterobacterales':ab,ti,kw 13095

#3 #1 OR #2 15067

#4 'avibactam plus ceftazidime'/exp 2426

#5 'avibactam-ceftazidime':ab,ti,kw OR 'ceftazidime-avibactam':ab,ti,kw OR 'avycaz':ab,ti,kw OR 'avibactam plus ceftazidime':ab,ti,kw OR 'nxl104':ab,ti,kw OR 'ave1330a':ab,ti,kw OR 'caz-avi':ab,ti,kw 1767

#6 #4 OR #5 2735

#7 'polymyxin'/exp 8690

#8 'polymyxin':ab,ti,kw OR 'polymyxin b':ab,ti,kw OR 'polymyxin m':ab,ti,kw OR 'colistin':ab,ti,kw OR 'polymyxin e':ab,ti,kw OR 'colimycin':ab,ti,kw OR 'colisticin':ab,ti,kw OR 'coly-mycin':ab,ti,kw OR 'totazina':ab,ti,kw OR 'colistin sulfate':ab,ti,kw OR 'sulfate, colistin':ab,ti,kw 20147

#9 #7 OR #8 26266

#10 #3 AND #6 AND #9 345

**Cochrane Library**

#1 carbapenem resistant Enterobacteriaceae 136

#2 (carbapenemase-Producing Enterobacteriaceae):ab,ti,kw OR (carbapenem resistant Klebsiella pneumoniae):ab,ti,kw OR (carbapenem resistant Escherichia coli):ab,ti,kw OR (Antibiotics, Carbapenem):ab,ti,kw OR (Carbapenem Antibiotics):ab,ti,kw OR (carbapenem resist):ab,ti,kw OR (carbapenemase producing):ab,ti,kw OR (carbapenem resistance):ab,ti,kw OR (carbapenem non-susceptible Enterobacteriaceae):ab,ti,kw OR (CNSE (Enterobacteriaceae)):ab,ti,kw OR (CRE (Enterobacteriaceae)):ab,ti,kw OR (carbapenem nonsusceptible enterobacterial isolate):ab,ti,kw OR (Carbapenem-Resistant Enterobacterales):ab,ti,kw 359

#3 #1 OR #2 383

#4 avibactam, ceftazidime drug combination 42

#5 (avibactam-ceftazidime):ab,ti,kw OR (ceftazidime-avibactam):ab,ti,kw OR (Avycaz):ab,ti,kw OR (avibactam plus ceftazidime):ab,ti,kw OR (nxl104):ab,ti,kw OR (ave1330a):ab,ti,kw OR (CAZ-AVI):ab,ti,kw 88

#6 #4 OR #5 93

#7 polymyxins 154

#8 (Polymyxin):ab,ti,kw OR (Polymyxin B):ab,ti,kw OR (Polymyxin M):ab,ti,kw OR (Colistin):ab,ti,kw OR (Polymyxin E):ab,ti,kw OR (Colimycin):ab,ti,kw OR (Colisticin):ab,ti,kw OR (Coly-Mycin):ab,ti,kw OR (Totazina):ab,ti,kw OR (Colistin Sulfate):ab,ti,kw OR (Sulfate, Colistin):ab,ti,kw 952

#9 #7 OR #8 1002

#10 #3 AND #6 AND #9 9
